# Supplementary material for: An insight into the causal relationship between sarcopenia-related traits and venous thromboembolism: A mendelian randomization study
Source: PLoS One. 2024 May 16;19(5):e0303148. doi: 10.1371/journal.pone.0303148 (PMC11098320; doi:10.1371/journal.pone.0303148)

Supplementary Figures

**An Insight into the Causal Relationship between Sarcopenia-Related Traits and Venous Thromboembolism: A Mendelian Randomization Study**

Figure S1. Scatter plots (a) and forest plots (b) from genetically predicted muscle weakness to VTE.


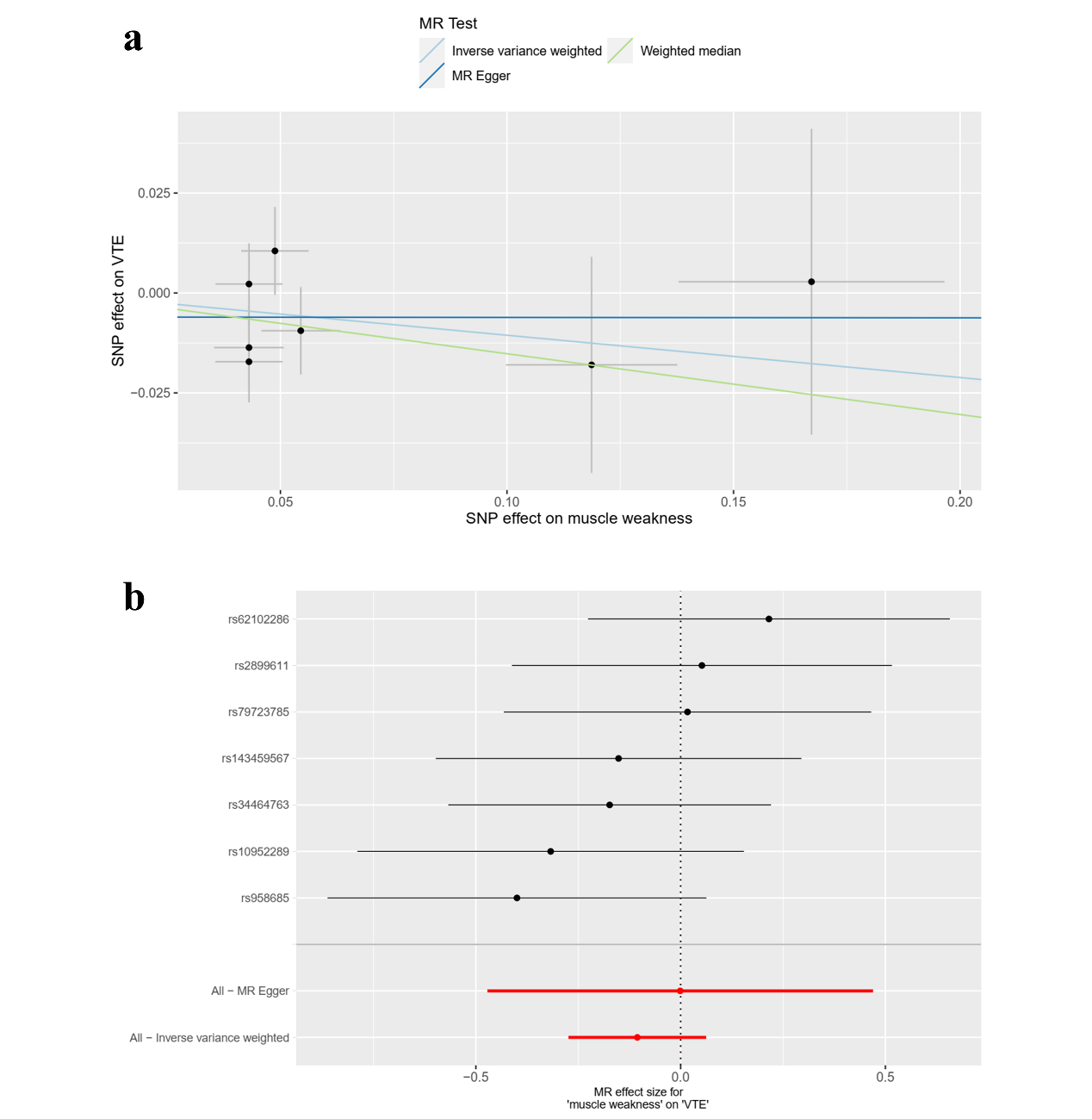


Figure S2. Leave-one-out analysis from genetically predicted muscle weakness to VTE.


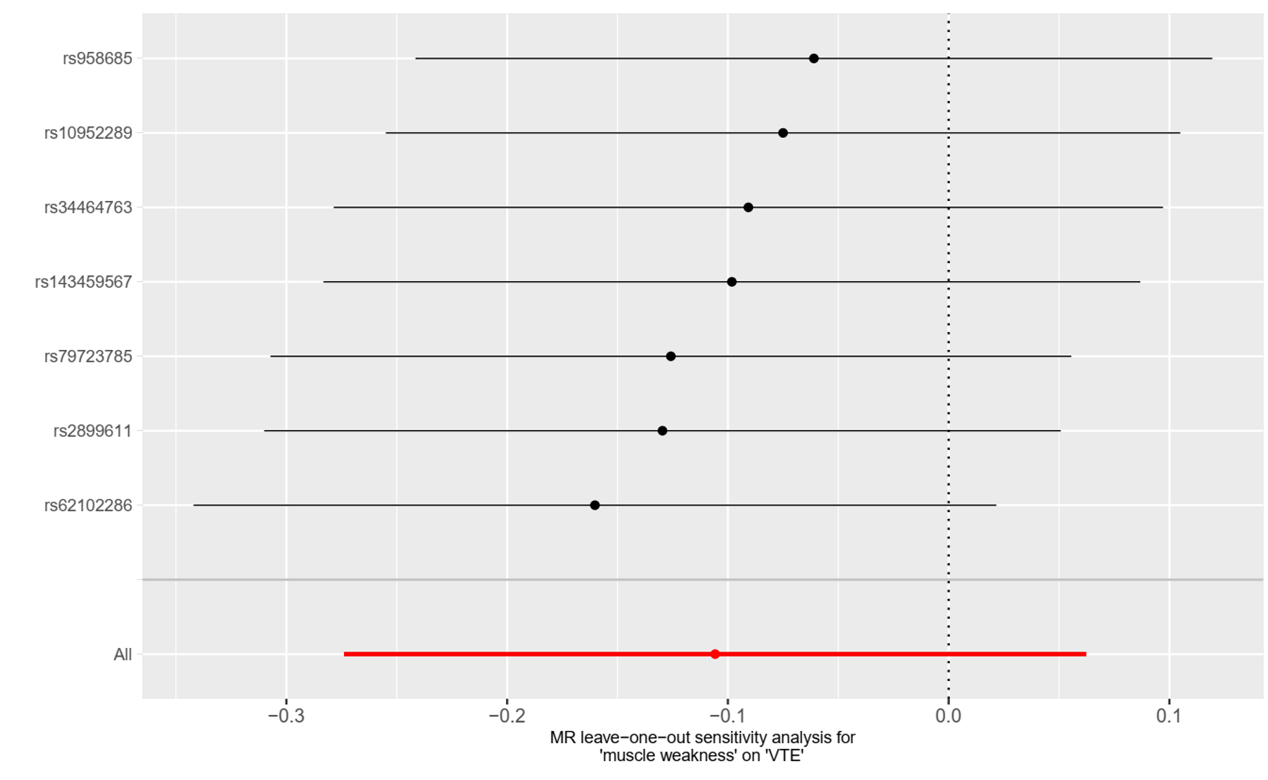


Figure S3. Funnel plot from genetically predicted muscle weakness to VTE.


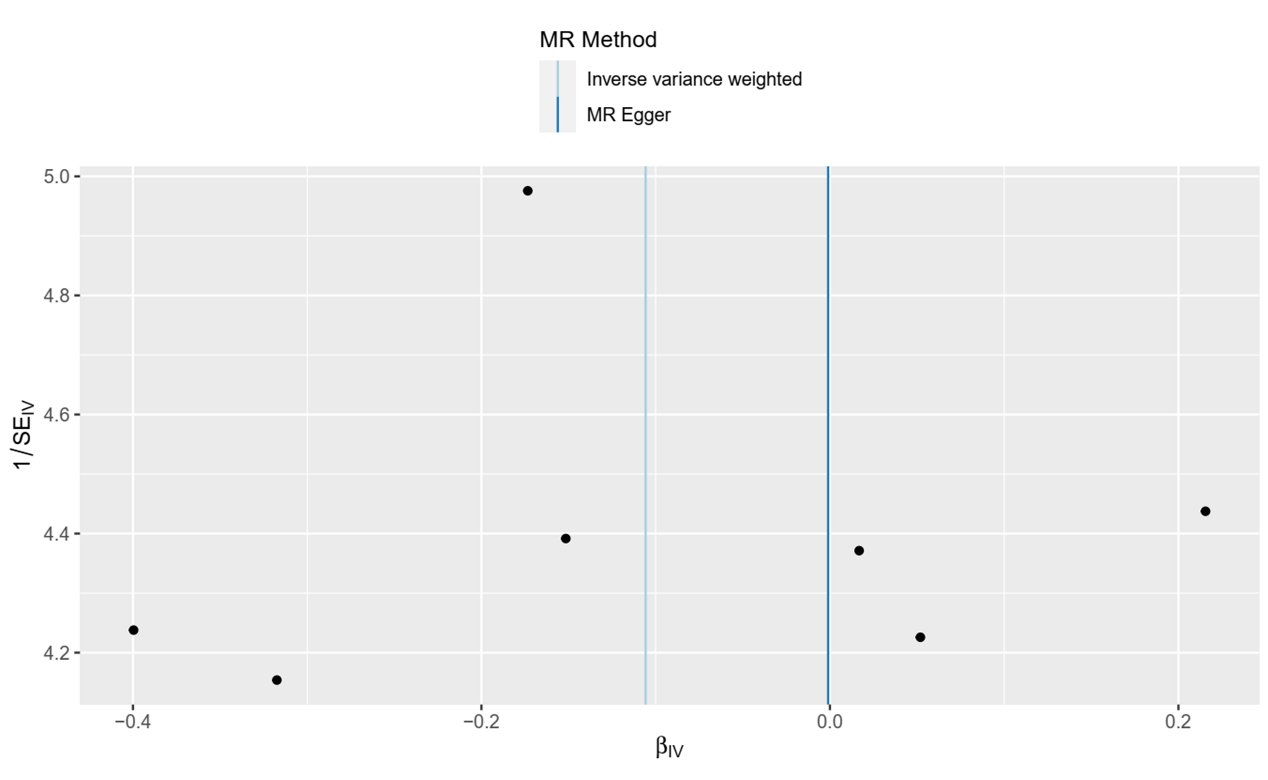


Figure S4. Forest plots from genetically predicted whole body lean mass to VTE.


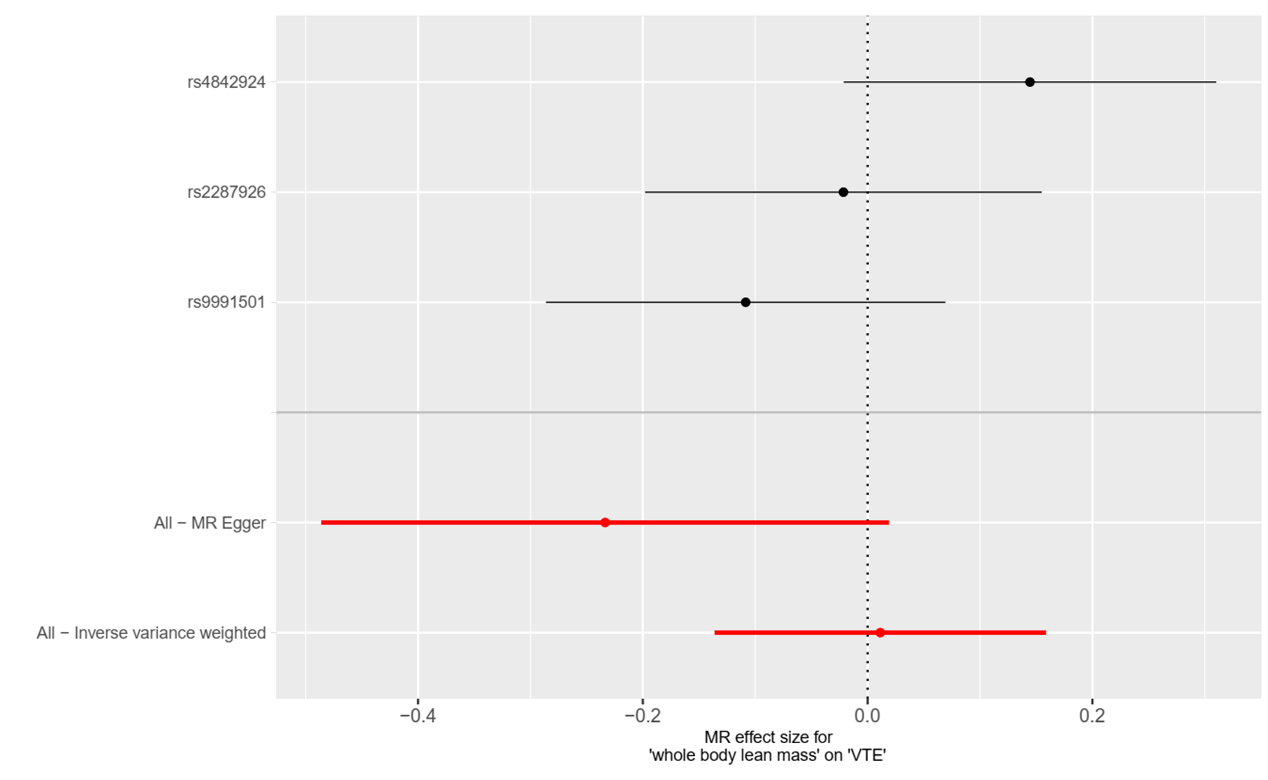


Figure S5. Leave-one-out analysis from genetically predicted whole body lean mass to VTE.
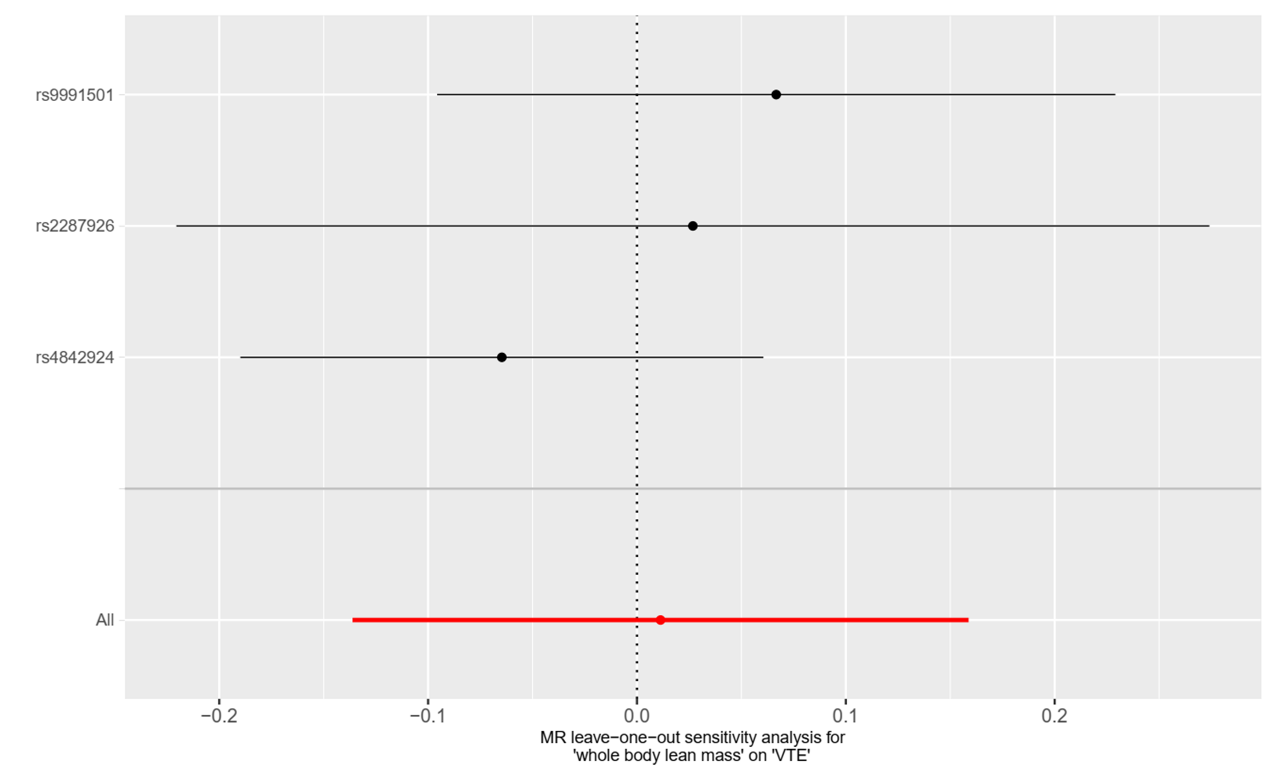

Supplement: S2 File — (DOCX) [file pone.0303148.s002.docx]
