# Supplementary material for: An insight into the causal relationship between sarcopenia-related traits and venous thromboembolism: A mendelian randomization study
Source: PLoS One. 2024 May 16;19(5):e0303148. doi: 10.1371/journal.pone.0303148 (PMC11098320; doi:10.1371/journal.pone.0303148)
Supplement: Attachment — Submitted filename: Comments_PONE-D-24-01767.docx [file pone.0303148.s003.docx]

Reviewer’s comments

This manuscript, “An Insight into the Causal Relationship between Sarcopenia-Related Traits and Venous Thromboembolism: A Mendelian Randomization Study”, by Du et al introduced a Mendelian Randomization (MR) study to assess the causal effect of sarcopenia on the risk of VTE. While the results show that the SNPs genetically predicted sarcopenia did not causally influence VTE directly, this is a well-designed and interesting study. The paper was well written in general. I have several comments for the authors.

1. The secondary analyses of the VTE risk factors were not adjusted for multiple testing (Table 3). Please clarify that in the paper.
2. The method of tightening the p-values from different methods is vague. Please provide more explanations and illustrate the use in the results.
3. Minor comments:
   a. Figure 1. Please add the number of SNP used at each step.
   b. Section 2.4. The weight median and MR-Egger analyses are essentially sensitivity analyses but not complements.
