# Supplementary material for: An insight into the causal relationship between sarcopenia-related traits and venous thromboembolism: A mendelian randomization study
Source: PLoS One. 2024 May 16;19(5):e0303148. doi: 10.1371/journal.pone.0303148 (PMC11098320; doi:10.1371/journal.pone.0303148)
Supplement: Attachment — Submitted filename: Response to Reviewers.docx [file pone.0303148.s004.docx]

**A thank-you letter and responses to reviewers**

Dear Editor Bo Hu,

We would like to express our great appreciation to you and the volunteer reviewers.

Thank you for the review comments, each point of which is helpful, constructive and meticulous. Although time is so precious, you spend your time processing and reviewing this article for a rigorous publication, it is self-giving of you! Humanity has made inexhaustible progress in the knowledge of nature, just because of anyone like you who works just for science, not for interest. As the co-corresponding author, I feel gratified because our scrupulousness won us plaudits from you, regardless of whether the review comments were positive. I have a “stubborn” belief that process counts more than results and attitude toward honesty and meticulousness counts more than ability. I can read your valuable quality of meticulousness and erudition from the meticulous review comments. And we are very glad to perfect our manuscript with your valuable peer-review suggestion.

Thank you, you deserve it, all of you!

Yours sincerely,

Shengyuan Gu on behalf of the authors. (Zhiwei Yao and Shengyuan Gu as the co-corresponding authors)

Tips for an easy reading of the revision, are as follows:

Here below are the responses to the review comments. The statement, “in line *хх*- line *хх* on page *xx*”, only refers to the marked-up revised version, not the unmarked revised version. In this Word file, the words in blue represent the points that are raised by the academic editor and reviewers and that we have addressed. The words in green represent the points that are raised but that we have not revised—of course, we give our reasonable answer from our perspective.

**Part 1.**

Additional Editor Comments: This study was carefully conducted and the paper was well written. My main comment is regarding further clarifications needed for certain important methods, e.g., whether the analysis on the VTE risk factors was corrected for multiplicity.

Response: It is a very relevant concern on the statistics issues showing your erudition and rigorousness. As the same question to Comment 1 of Reviewer #2, you can reach our answer in the specific part below.

**Part 2.**

Reviewer #1: Title: An Insight into the Causal Relationship between Sarcopenia-Related Traits and Venous Thromboembolism: A Mendelian Randomization Study

2.1. General Comment:

The paper presents a meticulously executed Mendelian randomization study delving into the causal relationship between sarcopenia-related traits and VTE. The study design is robust, and the analyses are conducted with a high level of rigor. These findings significantly contribute to our comprehension of the intricate interplay between sarcopenia, VTE, and associated risk factors. However, there are opportunities for minor enhancements such as providing effect sizes for indirect effects, discussing study limitations more comprehensively, and offering more specific recommendations for future research and clinical practice. Nevertheless, this study constitutes a substantial addition to the literature on sarcopenia and VTE.

Response: The two points in the above general comment (in blue) are scattered in the specific comment below and our answer is also in the specific comment. To the first point (in green), we think it is a good suggestion to provide effect sizes for indirect effects. We did not find a causal relation between sarcopenia and VTE but we found a potential pathway—through diabetes mellitus and hypertension—that might link sarcopenia to VTE, which is as much as to say that sarcopenia has not any direct effects on VTE and the total indirect effect size is 100% if the potential mediation pathway was real. From our perspective, it is more meaningful to calculate the indirect effect sizes on the premise that sarcopenia has a direct effect on VTE. Unfortunately, we have not found a direct effect of sarcopenia on VTE. And so, we did not revise our manuscript to this point.

2.2. Comment on Abstract:

The abstract succinctly outlines the study's objective, methodology, key findings, and conclusions, effectively emphasizing the knowledge gap concerning the causality between sarcopenia and VTE. To enhance clarity and contextualize the study's significance, incorporating a brief discussion on the prevalence and clinical significance of sarcopenia and VTE would be beneficial. Additionally, a minor grammatical error is noted in the last sentence of the conclusion, where "did not" should be replaced with "does not" to ensure consistency in verb tense.

Answer: To enhance clarity and contextualize the study's significance, we incorporate a brief discussion on the prevalence and clinical significance of sarcopenia and VTE in line *5* - line *8* on page *4*. We have corrected the grammatical error in line *2* on page *5* and in line *22* on page *16*.

2.3. Comment on Methods:

The methods section is meticulously detailed and well-organized, providing clear explanations of the study design, genetic variant selection, data sources, and statistical analyses. The rationale behind the selection of sarcopenia-related traits, genetic variants, and outcome measures is well-justified. Commendably, sensitivity analyses are employed to assess the robustness of the results.

The sentence "After harmonization of the effect alleles between sarcopenia-related traits and VTE and removal of the VTE-related SNPs (at a level of p < 5 × 10−8)" could be improved to "After harmonizing the effect alleles between sarcopenia-related traits and VTE and removing the VTE-related SNPs (at a significance level of p < 5 × 10−8)".

While the paper provides a clear explanation of the MR methods used, including IVW, weighted median, and MR-Egger analyses, further elaboration on why these specific methods were chosen and how they complement each other in addressing potential biases could be beneficial.

Answer: We have improved our expression as the kind reviewer suggested in lines *5* – *7* on page *8*. We have added further elaboration on why these specific methods were chosen and how they complement each other in addressing potential biases in lines *13* – *19* on page *8* and linked two references [*18, 19*] to help readers understand deeper and better on this issue.

2.4. Comment on Discussion and Conclusion:

2.4.1. Interpretation of Findings: The discussion section meticulously interprets the study's findings, particularly emphasizing the absence of direct causal evidence between sarcopenia-related traits and VTE. However, delving deeper into potential mechanisms underlying the observed indirect effects of sarcopenia on VTE through diabetes mellitus (DM) and hypertension (HTN) would strengthen this section. Offering insights into the biological pathways or physiological processes linking sarcopenia-related traits to these risk factors and ultimately to VTE would enhance the discussion.

Answer: We have given an insight into the potential mechanisms underlying the observed indirect effects of sarcopenia on VTE through diabetes mellitus (DM) and hypertension (HTN), and the biological pathways or physiological processes linking sarcopenia-related traits to these risk factors and ultimately to VTE in lines *2* - *13* on page *15*.

2.4.2. Implications and Future Directions: The conclusion effectively summarizes the study's key findings and highlights the potential indirect effects of sarcopenia on VTE through DM and HTN. Expanding on the implications of these findings for clinical practice and future research directions would provide a more comprehensive closure to the paper. Discussing how these results could inform preventive strategies or interventions targeting sarcopenia-related traits to reduce the risk of VTE could add practical value to the study.

Answer: We have expanded on the implications of these findings for clinical practice and future research directions and discussed how these results could inform preventive strategies or interventions targeting sarcopenia-related traits to reduce the risk of VTE in line *22* on page *15* and in lines *1* - *4* on page *16* and in lines *1*- *2* on page 17.

**Part 3.**

Reviewer #2:

3.1. General Comment: This manuscript, “An Insight into the Causal Relationship between Sarcopenia-Related Traits and Venous Thromboembolism: A Mendelian Randomization Study”, by Du et al introduced a Mendelian Randomization (MR) study to assess the causal effect of sarcopenia on the risk of VTE. While the results show that the SNPs genetically predicted sarcopenia did not causally influence VTE directly, this is a well-designed and interesting study. The paper was well written in general. I have several comments for the authors.

3.2. Comment: The secondary analyses of the VTE risk factors were not adjusted for multiple testing (Table 3). Please clarify that in the paper.

Answer: As many previous published MR studies have been done, we have not adjusted the p-value threshold for multiple testing in the risk factor analyses. We have clarified this issue in line *22* on page *9* and in line *1* on page *10*. Additionally, we also discuss it as a limitation in lines *12* - *18* on page *16*.

3.3. Comment: The method of tightening the p-values from different methods is vague. Please provide more explanations and illustrate the use in the results.

Answer: We have provided more explanations and illustrated the use in the results in lines *14* – *16* on page *11*.

3.4. Comment: Minor comments:

3.4.1. Comment: Figure 1. Please add the number of SNP used at each step.

Answer: Thank you for your useful advice. It is a very good idea to add the number of SNP used at each step, which would make our figure more detailed. After adding the number of SNP used at each step in Figure 1, we found the aesthetic degree of the figure become poor. We have made several attempts to achieve it, but unfortunately, we failed. To keep an aesthetical view of the figure, we would prefer to hold the figure staying the same.

3.4.2. Comment: Section 2.4. Section 2.4. The weight median and MR-Egger analyses are essentially sensitivity analyses but not complements.

Answer: We have revised it in lines *11* – *12* on page *8*.
